# Learning from the implementation phase of the new French capitation payment model for chronic kidney disease care: a qualitative study

**DOI:** 10.1007/s40620-025-02284-8

**Published:** 2025-04-22

**Authors:** Maxime Raffray, Arnaud Campéon, Damien Bricard, Estelle Augé, Denis Raynaud, Cécile Couchoud, Luc Frimat, Sahar Bayat

**Affiliations:** 1 CNRS, Inserm, Arènes–UMR 6051, RSMS–U1309, École des hautes études en sante publique, Rennes, France; 2 Clinical Epidemiology Division, Department of Medicine Solna, Karolinska Institutet, Maria Aspmans Gata 22A, 171 64 Solna, Sweden; 3 Arènes, CNRS, UMR 6051, École des hautes études en sante publique, Rennes, France; 4 Institut de Recherche et Documentation en Économie de la Santé, Paris, France; 5 Agence de la Biomédecine, Registre REIN, Saint-Denis, France; 6 Néphrologie, CHU de Nancy-Hôpitaux de Brabois, Vandœuvre-lès-Nancy, France

**Keywords:** Chronic kidney disease, Health policy, Capitation payment, Multidisciplinary care

## Abstract

**Background:**

France introduced a new payment model for care providers of patients with Chronic Kidney Disease (CKD) Grades 4 and 5: the CKD-Capitation Payment model. The model aims to financially incentivise multidisciplinary care for patients. We performed a qualitative study among participating providers to identify obstacles and facilitators of the model implementation as well as the initial benefits and potential policy improvements.

**Methods:**

From March to July 2023, we collected data through semi-structured interviews with medical and managerial staff of facilities participating in the new model in France. We purposely selected a sample of facilities based on ownership status and CKD-Capitation Payment model activity data, including the number of patients reported. We performed a thematic analysis of the interview transcripts.

**Results:**

We interviewed 22 staff from 14 facilities. Interviews revealed that adapting the information systems to the model requirements was a major obstacle to implementation, undermining efficient medical time allocation and data quality. Securing facility management support and organising the care amid workforce shortages were additional obstacles. Despite these challenges, staff reported positively on the model, noting the increased time spent by nurses with patients and the assertion of dietitians’ role. Interviewees reported the need for greater flexibility in visit requirements to better align with patient needs.

**Conclusions:**

This study demonstrates how the new capitation payment model introduced in France can enable multidisciplinary and coordinated care for patients with CKD. However, supporting facilities in adopting interoperable information systems and increasing the flexibility of the model appear essential for long-term adoption.

**Graphical abstract:**

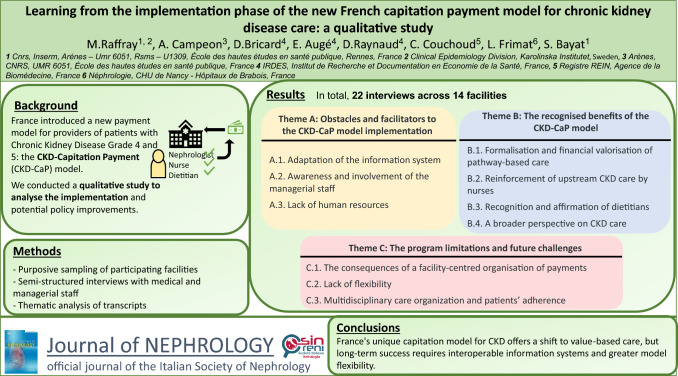

**Supplementary Information:**

The online version contains supplementary material available at 10.1007/s40620-025-02284-8.

## Introduction

Chronic Kidney Disease (CKD) is an important public health issue, with an estimated worldwide prevalence of 10% [1]. Disease progression to kidney failure requiring kidney replacement therapy (KRT) is an important and growing burden for patients [2] and healthcare systems, with substantial economic costs [3].

Several payment reforms have been deployed internationally to incentivise high quality and cost-effective care for CKD, with a strong focus on KRT [4]. Those reforms aim to financially incentivise home dialysis and kidney transplantation, such as the Best Practice Tariffs in the UK [5] or ESKD Treatment Choices Model in the US [6]. Upstream CKD care is only a more recent focus for payment reforms [7]. The US Kidney Care First (KCF) option is one example that introduced, in 2022, capitated payments to nephrology practices for managing the care of patients with CKD Grades 4 (G4) and 5 (G5), with adjustments on outcomes and quality measures [6].

In 2020, France launched a similar, yet different payment model for care providers targeting patients with CKD G4 and G5: the CKD-Capitation Payment (CKD-CaP) model. In France, the prevalence of KRT concerned 1362 individuals per million population (92,535 patients) in 2021 [8] and represented 4.4 billion € in annual health expenditure [9]. Additionally, emergency dialysis start represents 25% of incident dialysis starts each year [8–10]. The objectives of the CKD-Capitation Payment model are to financially incentivise multidisciplinary care for patients, delay CKD progression and improve the transition to KRT [11].

The aims of this paper are to 1) present the new payment model introduced in France and 2) analyse the implementation phase through the perspectives of medical and managerial staff of participating facilities. More precisely, we sought to address the following questions: What are the obstacles and facilitators of the new model implementation? what are the perceived initial benefits for the care and the potential improvements of the model?

## Methods

### The French healthcare system setting

The French healthcare system covers its population with statutory health insurance through social security, leading to very low average out-of-pocket expenses [12]. Furthermore, France offers complete coverage for the expenses of several chronic conditions, including CKD. Care is delivered by public (university and non-university hospitals) and private providers (hospitals or private clinics for profit and/or non-profit, self-employed health professionals). The dominant payment to providers in France is fee-for-service for outpatients, including visits and prospective activity-based funding, including case-mix for inpatient care for hospitals [12].

### The CKD-capitation payment model

The French CKD-Capitation Payment model launched in 2020 introduces the possibility for providers (excluding self-employed health professionals) to opt-in and receive an annual payment per enrolled patient (eligibility is conditional on ≥220 patient size). The target population includes patients with CKD G4 and G5 non-treated by dialysis (estimated Glomerular Filtration Rate [eGFR] = 15-29 and <15 ml/min per m^2^, respectively). In 2024, the amount varied between 319 € and 692 € per patient based on CKD grade and provider ownership status [11]. It covers the outpatient visits to nephrologists and visits to paramedical professionals for therapeutic education and disease management (nurses and dietitians). Each provider is free to organise the CKD care pathway. However, the amount received per patient is modulated by the number of annual visits received with different professionals: at least one nephrologist visit must be provided to receive a minimal amount (33%), at least one visit with a nurse to receive an additional 33%, and at least one visit with a dietitian to receive the final additional 33%. The participating facilities must gather and send their CKD-Capitation Payment activity data each calendar year to the French National Technical Agency for Information on Hospital care (ATIH) to receive the corresponding payment. Some data are mandatory to receive payment (e.g. number of visits), some are not (e.g. patient’s comorbidities, quality indicators).

### The qualitative study of the implementation of the model

We conducted a descriptive qualitative study based on semi-structured interviews with medical and managerial staff of facilities that opted-in the CKD-Capitation Payment model. The study focused on the obstacles and facilitators of implementation as well as the changes introduced.

We used a purposive sampling method to select facilities. We considered several characteristics that might be associated with differences in the model implementation: ownership status, number of patients enrolled, and proportion of patients who had received at least one visit with a nurse and dietitian. We selected 14 facilities with the aim of maximising variation across these characteristics using the centralised CKD-Capitation Payment data from all participating facilities (N=408 facilities, February 2023). The French School of Public Health (EHESP, co-author MR and SB) is authorised to access data centralised by the ATIH for research and teaching purposes. In the rationale of the CKD-Capitation Payment model, the nephrologists are presented as the key actors, e.g. they are the only professionals (and nurses and dietitians are crucial actors in kidney care delivery) whose visit is a requirement to receive any payment. Thus, they were considered the entry point in our selection of facilities. Whenever possible, or when the point of view of the nephrologist was deemed not sufficient to capture the situation at a given facility, we aimed at including other participants holding various positions (directors, dietitians, healthcare executives, nurses). Between March and July 2023, one of the authors (M.R., PhD, post-doc at the time with previous qualitative methods training) contacted and interviewed staff members at each of the selected facilities. Interviews were semi-structured with a guide (M.R., A. C., senior researcher in sociology) who retraced the CKD-Capitation Payment model implementation, the obstacles encountered and the changes introduced from the perspectives of the interviewee (interview guide available in Supplementary File A). After receiving the interviewees’ consent, M.R. recorded all interviews. During the interviews, M.R. took notes and reviewed them shortly after the end of the interview. Recruitment and interviews stopped once data saturation was reached, that is, the point when additional data did not lead to any new themes [13].

From the corpus of the interview transcripts, M.R. carried out a thematic content analysis [14]. In this method, the researcher becomes familiar with the data by first reading the transcriptions in their entirety, systematically reporting the information provided by each interview. The researcher then reads the transcriptions again, associating their content with as many themes as necessary to describe all their significant aspects and then merges all identified themes into a final list of analysis themes. We carried out the thematic content analysis using the NVivo 12 software. We followed the Consolidated criteria for reporting qualitative research (COREQ) guidelines for qualitative research using interviews (Supplementary File B).

## Results

**Fig. 1:**
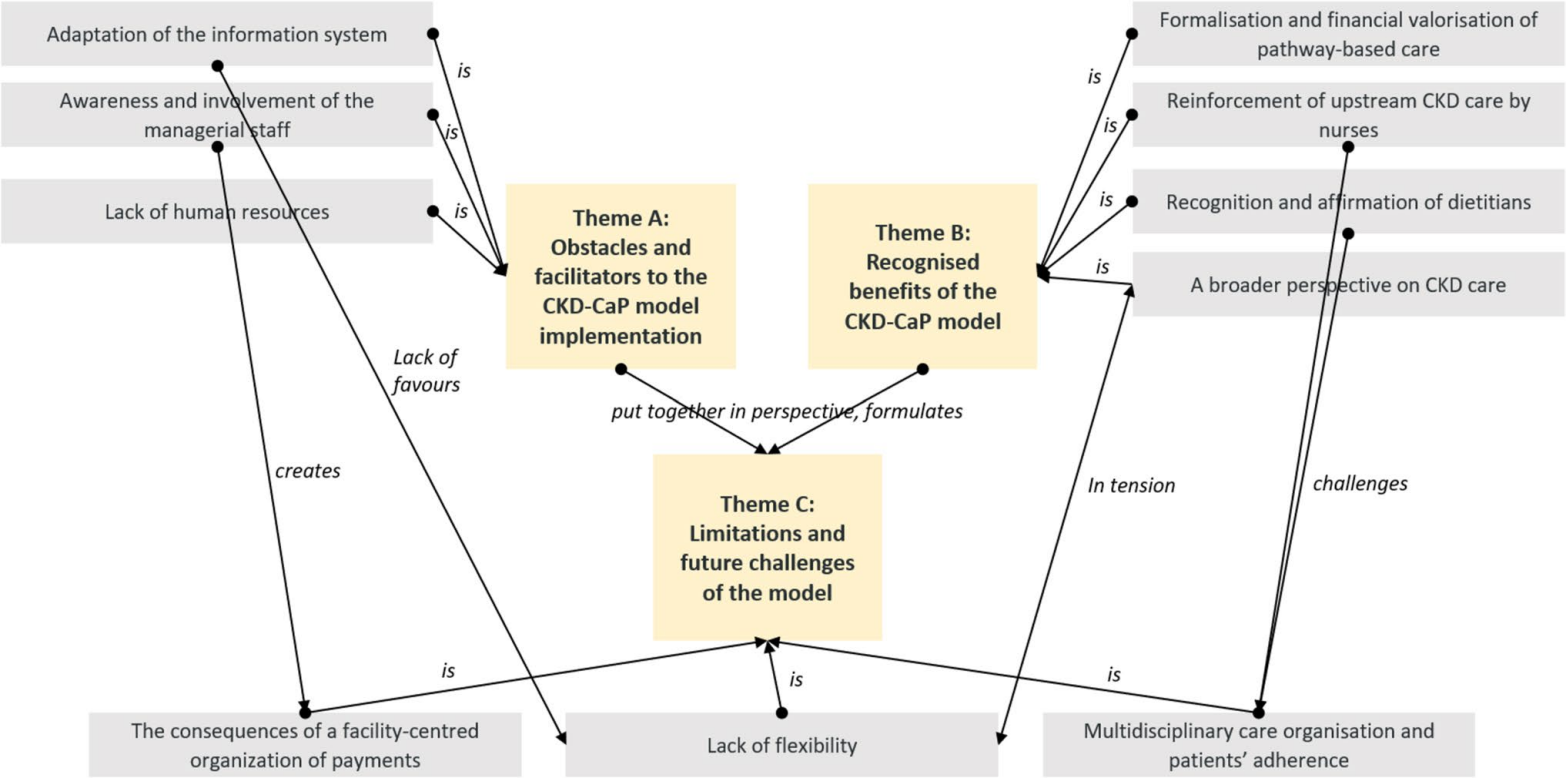
Summary of the themes identified (22 interviews with medical and management staff, 14 facilities)

Table 1 describes the characteristics of the 14 facilities sampled. We carried out 22 semi-structured interviews. The interviewees were 12 nephrologists, 4 directors, 2 healthcare executives, 3 dietitians, and 1 nurse (mean interview duration: 48 minutes). The thematic content analysis of the interview transcripts yielded three main themes and eleven sub-themes summarised in Figure 1. Boxes 1, 2, 3 present the supporting quotes from the transcripts of the interviews.

**Table 1:** Characteristics of the facilities selected for the semi-structured interviews

Facility	Ownership status	Activity description	French region	Number of patients enrolled in the CKD-CaP model in 2021	% of patients with ≥ 1 consultation with a nurse in 2021	% of patients with ≥ 1 consultation with a dietitian in 2021	Interviewees
A	Private non-profit	CKD only, with outpatient visits and dialysis	Auvergne-Rhône-Alpes	225	78.2	77.8	1 nephrologist, 1 director, 1 healthcare executive
B	Public	Teaching regional hospital, nephrology department	Grand Est	504	48	72.2	1 nephrologist, 1 healthcare executive
C	Public	Teaching regional hospital, nephrology department, outpatient and inpatient, dialysis	Overseas	186	68.8	59.7	1 nephrologist
D	Public	Hospital, nephrology department, outpatient and inpatient, dialysis	Hauts-de-France	313	0	0	1 nephrologist
E	Private non-profit	CKD only, outpatient visits and dialysis	Nouvelle Aquitaine	345	8.4	38.6	1 nephrologist, 1 nurse, 1 dietitian, 1 director
F	Public	Teaching regional hospital, nephrology department, outpatient and inpatient, dialysis	Ile-de-France	353	0	0	1 nephrologist, 1 director
G	Public	Hospital, nephrology department, outpatient and inpatient	Provence-Alpes-Côte d'Azur	41	63.4	63.4	2 nephrologists
H	Private non-profit	CKD only, outpatient visits and dialysis	Loire-Atlantique	609	66.8	67	1 dietitian
I	Public	Teaching regional hospital, nephrology department, outpatient and inpatient, dialysis	Grand Est	695	100	69.8	1 nephrologist
J	Private for-profit	Hospital, CKD focussed, outpatient visits, inpatient, dialysis	Occitanie	789	83.4	80.2	1 director
K	Private for-profit	Hospital, CKD focussed, outpatient visits, inpatient, dialysis	Nouvelle Aquitaine	675	58.4	52.9	1 dietitian
L	Public	Teaching regional hospital, nephrology department, outpatient and inpatient, dialysis	Auvergne-Rhône-Alpes	730	1.2	0	1 nephrologist
M	Private for-profit	Hospital, CKD focussed, outpatient visits, inpatient, dialysis	Haut-de-France	843	9.4	10.2	1 nephrologist
N	Public	Hospital, nephrology department, outpatient and inpatient, dialysis	Haut-de-France	184	20.1	19.6	1 nephrologist

### Theme A: The obstacles and facilitators to the CKD-Capitation Payment model implementation

#### Sub-theme A1. Adaptation of the information system

For billing, the CKD-Capitation Payment model requires data that were previously not recorded, such as visits with nurses and dietitians. Facilities were not prepared for this and adjusting the information system to the expected format was the most frequent difficulty reported. At some facilities, the electronic Health Records (eHR) system allowed extracting the required data, provided that a new "tab" in that system was created (Facilities B, I, L). At other facilities, the eHR system did not allow this change due to software inflexibility and lack of support from publishers, and required the creation of an ad-hoc spreadsheet (quote 1a). The search for information, its verification, and transfer to the computer tool required the reallocation of tasks typically performed by a clinical research assistant to nurses and secretaries. This was a significant investment by the staff at the expense of the time dedicated to patients that led to questioning the data usefulness and purpose (quotes 1b, 1c).

#### Sub-theme A2. Awareness and involvement of the managerial staff

The importance of the potential revenues from the CKD-Capitation Payment model was not evident to the managerial staff and in some cases was underestimated. This was compounded by the novelty of the model and the complexity of adapting the information system. It was especially observed at general hospitals where CKD care was a drop in an ocean of activities (quotes 1d, 1e). One interviewee at a private non-profit facility, in which CKD care was the only activity, reported a more proactive commitment from the managerial staff, with a deliberate orientation of the revenues from dialysis activity to support the model development (quote 1f). The capacity for dialogue between managerial and medical staff was a facilitating factor to implement and run the model. The co-realisation of an initial cost-benefit analysis helped raise awareness and promoted management’s support for the acquisition of human resources.

#### Sub-theme A3. Lack of human resources

The issue of human resources shortage was central for the model implementation and running. Many interviewees stressed that nurses were a scarce resource (quote 1g) and subject to significant turnover. Although the difficulty of recruiting and retaining nurses was a general problem, it was particularly crucial in CKD care because of the training and specialisation time required.

#### Sub-theme A4. COVID-19 pandemic

The CKD-Capitation Payment model was launched in 2020 during the COVID-19 pandemic. The interviewees reported that the pandemic had, overall, a limited impact on the implementation, with the exception of the acute phases (and related lockdown periods). Indeed, remote consultations were provided to patients and remote meetings related to the model implementation were organised (quote 1h).

### Theme B: Recognised benefits of the CKD-Capitation Payment model

#### Sub-theme B1. Formalisation and financial valorisation of pathway-based care

Despite the difficulties related to data collection, the model implementation allowed the staff to visualise and track the pre-model CKD care activity but also to use it as a basis to discuss practices (quote 2a). The formalisation of a pathway in which nurse and dietitian consultations are operational objectives rendered the care more systematic, ultimately harmonising practices within some facilities (quote 2b). Interviewees appreciated the financial valuation of pre-existing activity and perceived it as a recognition of the work undertaken.

#### Sub-theme B2. Reinforcement of upstream CKD care by nurses

Many interviewees reported that dialysis nurses were often transferred to the management of patients with CKD but not requiring dialysis, instead of recruiting new ones dedicated to this task. The resulting reinforcement of upstream CKD care was appreciated by both staff and patients (quote 2c). Their roles included screening and identifying the patients' needs (e.g. social difficulties, mental health), blood pressure monitoring, medication review, liaising with the general practitioner, and delivering and discussing pre-dialysis information.

#### Sub-theme B3. Recognition and affirmation of dietitians

The financial valorisation of visits with dietitians asserted the role of nutrition in CKD care. This was not entirely new for CKD, particularly for patients treated by dialysis. However, its extension to the pre-dialysis stages was considered an important benefit of the model. Interviewees emphasised the importance of translating and simplifying the messages from nephrologists related to diet (quote 2d).

#### Sub-theme B4. A broader perspective on CKD care

Along with the increase of the nurse’s and dietician’s time, some facilities further expanded the scope of care to physical activity and social needs, involving, for example, a social worker. Territorial planning of healthcare provisions benefited from placing new staff in peripheral, smaller facilities (quote 2e). Some interviewees reported partnerships with other facilities to promote better access to CKD care. Some of these initiatives came from facilities that had implemented the CKD-Capitation Payment model, while some came from non-eligible, smaller, facilities. However, the conclusion of partnerships was difficult, especially when there was an imbalance in the patient population size (quote 2f).

### Sub-theme C: Model limitations and future challenges

#### Sub-theme C1. The consequences of a facility-centred organisation of payments

In the CKD-Capitation Payment model, the payment to facilities is not ring-fenced to CKD services and staff, which created disparities in the visibility and acquisition of dedicated resources. These disparities varied according to facility size, ownership status and quality of communication between staff and management. The distinction between facility and staff appeared less pronounced in non-profit private facilities where all were united around a common cause, namely CKD. Conversely, negotiating resources from a shared pool with other services was more challenging in hospitals (quote 3a). Additionally, some interviewees reported sentiments of exclusion and frustration from non-facility-based providers (quote 3b).

#### Sub-theme C2. Lack of flexibility

At least one visit with a nephrologist, with a nurse and with a dietitian within a calendar year are required to receive complete payment for a patient. Some interviewees thought that this could introduce the risk of trying to meet this requirement without taking into account their relevance for the patient (quote 3c). Some interviewees reported a desire for flexibility regarding the choice of healthcare professionals to work along with the nephrologist, in order to financially value the alignment of care with the evolving needs of patients (quote 3d).

#### Sub-theme C3. Multidisciplinary care organisation and patient adherence

The model increased the number of professionals involved in CKD care, questioning their coordination and usefulness. Indeed, some interviewees reported that in the first year of enrolment, visits with a nurse and a dietitian had an added value for patients. However, in the next years, the patients’ perception was more nuanced, with expressed redundancy (quote 3e, 3f). Additionally, scheduling appointments with the different professionals on the same day to reduce the patients’ travel burden was a challenge and often discarded as an option.

## Discussion

This study presents an analysis of the implementation of a new payment model for upstream CKD (G4 and G5) care in France in an effort to shift from a fee-for-service model towards value-based care. Our study is the first to report on this new model. Overall, the model was positively welcomed by stakeholders, with reports of reinforced routine multidisciplinary care. However, we found several obstacles to its implementation: adaptation of information systems, awareness and involvement of the facilities management, and lack of human resources. We also identified the lack of flexibility of the model as a limitation to be addressed.

Multidisciplinary care is not routinely implemented in many countries in Europe [15, 16], despite positive results reported on CKD outcomes [17]. To our knowledge, the French CKD-Capitation Payment model has no direct equivalent in Europe. The nationwide scope as opposed to local initiatives and the use of a capitation payment modulated by the number of different healthcare professionals makes it quite unique. In Canada, multidisciplinary care has been organised at dedicated clinics since at least 10 years, employing dietitians, social workers and pharmacists. However, large differences in practices and funding across provinces do not facilitate comparisons with the French CKD-Capitation Payment model [18]. At the beginning of 2022, the US introduced new value-based payment models for CKD care: the Kidney Care Choices model with a variety of options [7, 19]. Besides the major differences in the USA and French healthcare systems, the French CKD-Capitation Payment model does not adjust the payments in function of patient complexity ( reporting comorbidities is mostly non-mandatory) or outcomes. However, this might change as the latest version of the model included a bonus payment for reporting healthcare quality and outcome indicators (i.e. pre-transplant evaluation) starting in 2023 [20].

The CKD-Capitation Payment model implemented in France is both a timely and relevant response to the contemporary challenges of improving CKD care. Indeed, a recent review by Bello et al. demonstrated significant gaps in oversight, funding, and infrastructure for kidney care in the world [21]. In particular, a substantial proportion of countries do not publicly fund the prevention of progression of kidney disease. In a recent joint statement, nephrology societies called for the World Health Organization (WHO) to recognise kidney disease as a major driver of non-communicable disease-related mortality [22]. They advocate, among others, for developing, testing and scaling up novel balanced models of care, including extending the range of services to non-specialised professionals in an integrated effort. The aim of the CKD-Capitation Payment model is to enable multidisciplinary and coordinated care within the hospital setting for patients with chronic kidney disease grades 4 and 5, ultimately aiming to prevent progression to replacement therapy. We argue that this model can be implemented in other countries, regardless of the specificities of their health systems (organisation and funding). Indeed, at its core, the model relies on low-entry level requirements, e.g. collecting the number of visits with the different professionals and patient data. However, our findings indicate that there are obstacles that need to be considered.

We highlighted the difficulty for facilities to collect and send the data required by the CKD-Capitation Payment model. The lack of adaptation of the information system was the main obstacle. It was compounded by the increased redundancy of information to input to different sources (e.g. patients’ characteristics for disease quality registers) and workforce shortage. In recent years, electronic Health Records system completion has been associated with increased documentation time by hospital staff [23] and is often cited as a key factor for dissatisfaction with work and burnout [24]. Although in our study, staff found new ways to carry out the mandatory data collection (e.g. ad-hoc spreadsheets), the capacity of facilities to adapt should not be overestimated at the risk of implementation failure [25]. Moving forward, it appears critical that solutions to accompany facilities in adopting interoperable information systems should be developed, in which the data would be produced once during routine care and be reusable with minimal effort. Doing so would ensure a) the efficient allocation of medical time, b) the quality of the data and c) the ability to evaluate the efficacy of interventions or experimentations, such as a new payment model.

We reported that the requirement of yearly consultations with a nurse and with a dietitian to receive the complete payment could lead to a feeling of redundancy by patients. This is in line with previous studies reporting patients’ experience of redundancy of information with two professionals (e.g. between nurses and nephrologists or between nephrologists and dietitian) [26] but also with a single professional (e.g. dietitian) [27]. Our results suggest that the one-size-fits-all nature of the model should evolve to expand the range of professionals it includes. This will financially value the alignment of care with the evolving needs of patients. For example, a US study found positive outcomes among older patients with G3-G5 CKD when they were seen by a nephrologist, dietitian social worker and pharmacist compared to being seen by a nephrologist alone [28]. In our study, facilities often moved dialysis nurses to upstream CKD care, acquiring very diverse roles. Despite being associated with positive outcomes in CKD care [29, 30], the development of nurse practitioners remains in its infancy in France (legislated in 2018, 581 nurse practitioners for 637,000 nurses in 2021 [31]). Indeed, in our study only 3/14 facilities where interviews were carried out had a nurse practitioner. Further research and discussion must be carried out on the nurse practitioners’ role in upstream CKD care, specifically their articulation with other nurses and also with general practitioners who are particularly involved in the care of patients with CKD G4 and G5 [32]. It is worth noting that the model was first implemented during the COVID-19 pandemic. The pandemic may have had impacts not measured or reported here. Specifically, context-driven priorities for regulatory bodies might have delayed the provision of recommendations for a common model of a multidisciplinary care pathway.

One limitation of our study is that we did not include facilities that were interested in the model but did not opt in (e.g. because of an insufficient active patient list, <220 patients), and facilities that opted out. Such interviews could have highlighted additional challenges to the model implementation and/or operation. Half of the interviewees were nephrologists. This may have led to a limited representation of other points of view in our findings, including those of nurses. Nurses are critical in the care of pre-dialysis patients and it is assumed that the success of new care models depends on them. We should mention that the points of view of nephrologists we collected also included indirect feedback from nurses. Nonetheless, the relatively low number of nurses interviewed (n=1) limits the depth of the description of the changes that occurred. As such, their input should be the object of a dedicated study.

The next step will be to assess the long-term outcomes of this model (e.g. kidney failure and kidney replacement therapy incidence, pre-emptive kidney transplant and emergency dialysis start rate) and its cost-effectiveness. By covering a large population, the CKD-Capitation Payment model has the potential to provide robust results regarding the extent of multidisciplinary care efficacy, including the promise of personalised care (i.e. which pattern of care works best for which patients). The CKD-Capitation Payment model could also indirectly improve comprehensive conservative care for patients with kidney failure through the resources deployed.

## Conclusion

This study shows that the new capitation payment model introduced in France can enable multidisciplinary and coordinated care for patients with chronic kidney disease. However, supporting facilities in adopting interoperable information systems and increasing the flexibility of the model appear essential for long-term adoption. Future research is needed to assess long-term outcomes and cost-effectiveness of this payment model.

Box 1. Theme A: Obstacles and facilitators to the CKD-CaP model implementation:

**Table:** 

1a. The eHR system is [name]; it is impossible to create anything. We could have created a file for all patients in the model with administrative, medical information, etc., to be taken from the medical record. But it takes years to do that, so we are told, "fill in the spreadsheet," and that's it. *Nephrologist, Facility F*
1b. There were entire evenings and days with my colleague and the secretaries who devoted themselves to do just that […]; we are depriving the patient of pure raw medical time… *Nephrologist, Facility G*
1c. The primary utility of that spreadsheet is for a facility to be remunerated based on its activity, no more, no less. A lot of data was added for collection that are interesting for researchers, for scientific societies, for the ministry to assess the relevance of its tool, but ultimately, it falls on the healthcare professionals who may not necessarily see or understand this logic.* Director, Facility J*
1d. In 2021, it represented €55,000 in revenue. However, when compared to the overall [nephrology] service activity, including hospitalizations, it's not much; it's €50,000 out of eight million. *Deputy Director of Finance, Facility F*
1e. It felt like they were doing me a favour by agreeing to do this […]. For 300 patients, the question of profitability shouldn’t arise. It is roughly the same volume at Facility X, maybe even a bit lower, and over there the hospital director said, "let's go for it right away." *Nephrologist, Facility F*
1f. I can tell you that the lump sum [of the model] is not sufficient. I don't think it even covers the personnel costs: working time of the nephrologist, dietitian, nurse, and the time of the medical secretary. This means that the revenue from dialysis will finance the shortfall […]. We use the resources we have from patients treated by dialysis for patients with upstream CKD. *Director, Facility E*
1g. This nurse divides her time between haemodialysis (50%) and the care of patients with CDK (50%). I would like to increase her time on CDK to 100%, to optimize the pathways. But for now, I lack human resources in haemodialysis, so I can't rob Peter to pay Paul. *Health manager, Facility A*
1h. It [COVID-19 pandemic] didn't prevent us from holding these project meetings […] Once the conditions in hospitals could ensure the safe arrival and care of patients, everything had to continue. *Health manager, Facility B*

Box 2. Theme B: The recognized benefits of the CKD-CaP model.

**Table:** 

2a. Having all these data to report makes us look at what we're doing. Now, I conduct an annual review of the activity that concerns all these patients, which I present in a meeting with the team […] by examining what we do, we inevitably ask questions and try to correct any deviations. *Nephrologist, Facility I*
2b. This has allowed us to systematize a number of things. Maybe in some cases, we thought, 'oh, is the dietitian really necessary?' or the nurse, so I think it has made these different steps more systematic. *Health Manager, Facility B*
2c. It is professionally rewarding when patients say, 'It's true that you use different words to explain things, that you are less in a hurry than the doctor.' In reality, we are not less in a hurry than the doctor; we take the time. *Nurse at Facility E*
2d. We have theoretical data, that is, a patient should consume 0.8 g of protein per kilogram per day. It doesn't really resonate with them. So, they need someone who won't tell them what they're not allowed to eat but will guide them on what they can put on their plate, which is quite different. *Nephrologist, Facility B*
2e. Now they also have dietary care nearby [peripheral facility]. That's a real plus, decreasing travel times that could be a deterrent for some patients to come and this thanks to the model. *Dietitian, Facility E*
2f. We were approached by medium-sized facilities because we can enter into agreements to collectively reach the threshold [eligibility at 220 patients]. In this case, it was Facility Y, but for them… well, it's a bit annoying because, if we do that, it means we have to report their activity […] it's quite a hassle for what is essentially peanuts, I want to say. So, we weren't very enthusiastic. *Deputy Director of Finance, Facility F*

Box 3. Theme C: The model limitations and future challenges

**Table:** 

3a. I thought that if we showed that we were bringing in a lot of money—and there I thought 400,000 euros is not bad at all—we could say: 'we want this,' while being reasonable. I was told to write a project. I have many responsibilities, and writing a ten-page project to say what I do is simply too much. *Nephrologist, Facility L*
3b. I got a bit upset with some general practitioners in the area. They wanted us to come and talk about CKD. And there was an outcry: ‘we must stop thinking that only the hospital does it, we also want to implement therapeutic education outside the hospital, we have an advanced practice nurse who was trained in therapeutic education.' *Nephrologist, Facility D*
3c. We do an enormous amount of work towards the end of the year, we think, 'oh dear, this patient hasn't seen the dietitian, hasn't seen the nurse,' and we chase after the patients. So, that is also stressful and very time-consuming. *Health Manager, Facility A*
3d. You have patients for whom indeed, dietary management is fundamental, but you have others for whom that's not the core issue. And maybe patients for whom an initial psychological evaluation or a debriefing moment with a psychologist would be more appropriate. I find it a bit unfortunate because there might be patients for whom we bring in professionals when the need arises but is no longer there or didn't exist initially, and for whom we could bring in other skills… we don't set the pace, it's the illness. *Director, Facility J*
3e. The follow-up with the coordinating nurses is a bit complicated. Especially because patients who have been followed for a long time and have already had contacts with the therapeutic education nurse, the transplantation coordinating nurse, and our advanced practice nurses. So, they feel like they see 36,000 nurses, and they struggle to make a distinction. *Nephrologist, Facility I*
3f. The first year, there's no issue, patients come. And then when they are re-invited the next year, they say, 'actually, I already got the information.' So, some say, 'no, I don't want to come back for this'. *Nephrologist, Facility L*

## Supplementary Information

Below is the link to the electronic supplementary material.

Supplementary file1 (PDF 257 kb)

Supplementary file2 (PDF 480 kb)

## Data Availability

The ethics approvals prevent sharing the data, including participants' interviews transcripts.

## Abbreviations

ATIH: French National Technical Agency for Information on Hospital care
CKD: Chronic kidney disease
eHRs: Electronic health records
KRT: Kidney replacement therapy
CKD-CaP: Chronic kidney disease capitation payment
FFS: Fee for service
