# Supplementary material for: Learning from the implementation phase of the new French capitation payment model for chronic kidney disease care: a qualitative study
Source: J Nephrol. 2025 Apr 22;38(7):1877–87. doi: 10.1007/s40620-025-02284-8 (PMC12484326; doi:10.1007/s40620-025-02284-8)
Supplement: Supplementary file 1 — Supplementary file1 (PDF 257 kb) [file 40620_2025_2284_MOESM1_ESM.pdf]

Supplementary Files for "*What can we learn from the implementation phase of the new French capitation payment model for CKD G4 and G5: a qualitative study*"

Supplementary file A. Interview Guide

Supplementary file B. COREQ (COnsolidated criteria for REporting Qualitative research) Checklist

<u>Supplementary file A. Interview Guide</u>

Implementation of the CKD-CaP model

Interview guide

Presentation

How long have you been *practicing as a nephrologist*? [ADAPT]

In which healthcare facilities are you currently practicing?

How many patients do you see in consultation each year? (Total consultation activity)

- Among them, how many patients with chronic kidney disease (CKD) not on dialysis do you see each year? (rough estimate)

A. CKD care organization before the CKD-CaP model implementation

Can you describe the CKD pathway organization at your healthcare facility?

- When was the CKD pathway put in place at your facility?
- How many nephrologists, paramedics... (before/after the CKD-CaP model implementation)?
- Does the facility have connections for CKD care with other structures or healthcare providers?

Before the implementation of the CKD-CaP model, how was the follow-up of a patient with CKD G4/G5 organized in your service/facility?

- Does the population seen at your facility have specific characteristics? (e.g. socioeconomic?)

Have you participated in experimental models related to CKD care?

- What do you think of them?

B. Implementation of CKD-CaP model

Can you describe how the CKD-CaP model implementation was organized? (dates)

- The first time you heard about it?
- Were there any difficulties?

The model requires the collection of specific data, how was the information system organized to do this?

- How is data transmission organized today to receive the payment every year?

Whom did you collaborate with to implement the model?

- Are these new collaborations?
- Who are the patients included in the model?
- How many patients? Are those all the patients with CKD G4/G5 followed at your facility?
- What about patients with CKD G3b, transplant recipients?
- Do you know the yearly amount you receive? (visibility/resources)

C. Effects of the model

What has the model allowed to set up for the follow-up of patients with CKD G4/G5?

- Today, what is the typical care pathway for a patient with CKD G4/G5 that you follow?
- What are the new/additional resources?

For you, as a healthcare professional, what has the model brought?

- Do you observe changes in your daily practice?

Do you observe new collaborations?

- Within your facility?
- With primary care actors?
    - What is the general practitioner's role in the follow-up of patients with CKD G4/G5?

Has the model led to other changes?

D. The future of the CKD-CaP model

For you, what are or were the objectives of the package?

- Have these objectives been achieved in your facility, at your level?

What assessment do you make of the model?

- Are there changes you would like to see happen? What are the difficulties that persist in CKD care?

Supplemental File B.
COREQ (COnsolidated criteria for REporting Qualitative research) Checklist

A checklist of items that should be included in reports of qualitative research. You must report the page number in your manuscript where you consider each of the items listed in this checklist. If you have not included this information, either revise your manuscript accordingly before submitting or note N/A.

Topic	Item No.	Guide Questions/Description	Reported on Page No.
**Domain 1: Research team and reflexivity**			
Personal characteristics			
Interviewer/facilitator	1	Which author/s conducted the interview or focus group?	5
Credentials	2	What were the researcher's credentials? E.g. PhD, MD	5
Occupation	3	What was their occupation at the time of the study?	5
Gender	4	Was the researcher male or female?	N/A
Experience and training	5	What experience or training did the researcher have?	N/A
Relationship with participants			
Relationship established	6	Was a relationship established prior to study commencement?	5
Participant knowledge of the interviewer	7	What did the participants know about the researcher? e.g. personal goals, reasons for doing the research	N/A
Interviewer characteristics	8	What characteristics were reported about the inter viewer/facilitator? e.g. Bias, assumptions, reasons and interests in the research topic	N/A
**Domain 2: Study design**			
Theoretical framework			
Methodological orientation and Theory	9	What methodological orientation was stated to underpin the study? e.g. grounded theory, discourse analysis, ethnography, phenomenology, content analysis	5
Participant selection			
Sampling	10	How were participants selected? e.g. purposive, convenience, consecutive, snowball	5
Method of approach	11	How were participants approached? e.g. face-to-face, telephone, mail, email	5
Sample size	12	How many participants were in the study?	5,6
Non-participation	13	How many people refused to participate or dropped out? Reasons?	N/A
Setting			
Setting of data collection	14	Where was the data collected? e.g. home, clinic, workplace	5
Presence of non-participants	15	Was anyone else present besides the participants and researchers?	5
Description of sample	16	What are the important characteristics of the sample? e.g. demographic data, date	6,table 1
Data collection			
Interview guide	17	Were questions, prompts, guides provided by the authors? Was it pilot tested?	5
Repeat interviews	18	Were repeat inter views carried out? If yes, how many?	N/A
Audio/visual recording	19	Did the research use audio or visual recording to collect the data?	5
Field notes	20	Were field notes made during and/or after the inter view or focus group?	5
Duration	21	What was the duration of the inter views or focus group?	6
Data saturation	22	Was data saturation discussed?	5
Transcripts returned	23	Were transcripts returned to participants for comment and/or	N/A

Topic	Item No.	Guide Questions/Description	Reported on Page No.
		correction?	
**Domain 3: analysis and findings**			
Data analysis			
Number of data coders	24	How many data coders coded the data?	5
Description of the coding tree	25	Did authors provide a description of the coding tree?	N/A
Derivation of themes	26	Were themes identified in advance or derived from the data?	5
Software	27	What software, if applicable, was used to manage the data?	5
Participant checking	28	Did participants provide feedback on the findings?	N/A
Reporting			
Quotations presented	29	Were participant quotations presented to illustrate the themes/findings? Was each quotation identified? e.g. participant number	19-21
Data and findings consistent	30	Was there consistency between the data presented and the findings?	12
Clarity of major themes	31	Were major themes clearly presented in the findings?	6-10
Clarity of minor themes	32	Is there a description of diverse cases or discussion of minor themes?	6-10

Developed from: Tong A, Sainsbury P, Craig J. Consolidated criteria for reporting qualitative research (COREQ): a 32-item checklist for interviews and focus groups. *International Journal for Quality in Health Care*. 2007. Volume 19, Number 6: pp. 349 – 357
